# The current state of biomarker research for Friedreich’s ataxia: a report from the 2018 FARA biomarker meeting

**DOI:** 10.2144/fsoa-2019-0026

**Published:** 2019-06-28

**Authors:** Ian A Blair, Jennifer Farmer, Steven Hersch, Jane Larkindale, David R Lynch, Jill Napierala, Marek Napierala, R Mark Payne, Sub H Subramony

**Affiliations:** 1 Department of Systems Pharmacology and Translational Therapeutics Perelman School of Medicine, Penn SRP Center and Center of Excellence in Environmental Toxicology Center, University of Pennsylvania, Philadelphia, PA 19104, USA; 2 Friedreich’s Ataxia Research Alliance, 533 W Uwchlan Ave Downingtown, PA 19335, USA; 3 Voyager Therapeutics, Cambridge, MA 02139, USA; 4 Departments of Pediatrics and Neurology, Children's Hospital of Philadelphia, Philadelphia, PA 19104, USA; 5 Department of Biochemistry and Molecular Genetics, University of Alabama at Birmingham (UAB), 1825 University Boulevard, Birmingham, AL 35294, USA; 6 Department of Medicine, Division of Pediatrics, Indiana University, Indianapolis, Indiana, IN 46202, USA; 7 Department of Neurology McKnight Brain Institute, University of Florida, Gainesville, FL 32611, USA

**Keywords:** biomarkers, drug development, Friedreich’s ataxia

## Abstract

The 2018 FARA Biomarker Meeting highlighted the current state of development of biomarkers for Friedreich’s ataxia. A mass spectroscopy assay to sensitively measure mature frataxin (reduction of which is the root cause of disease) is being developed. Biomarkers to monitor neurological disease progression include imaging, electrophysiological measures and measures of nerve function, which may be measured either in serum and/or through imaging-based technologies. Potential pharmacodynamic biomarkers include metabolic and protein biomarkers and markers of nerve damage. Cardiac imaging and serum biomarkers may reflect cardiac disease progression. Considerable progress has been made in the development of biomarkers for various contexts of use, but further work is needed in terms of larger longitudinal multisite studies, and identification of novel biomarkers for additional use cases

## Lay abstract

Biomarkers are characteristics that can be objectively measured, evaluated and used as indicators of disease progression or the effect of a therapy. Friedreich’s ataxia is a progressive multisystem neuromuscular disease with no treatment. Current clinical measures cannot robustly detect disease progression in less than a year, meaning that clinical trials are long and drug development is slow. The Friedreich’s Ataxia Research Alliance and the scientific community are looking for biomarkers that show change in shorter time frames that can accelerate drug development. The 2018 FARA Biomarker Meeting summarized the exciting findings that represent the current state of the field.

Friedreich’s ataxia (FA) is a debilitating, life-shortening, degenerative neuromuscular disorder affecting approximately 1 in every 50,000 people in the USA and Europe [1]. Initial symptoms, typically seen in children and teenagers, include loss of balance and coordination and fatigue. As the disease progresses, patients develop dysarthria, vision and hearing loss, and many develop cardiomyopathy and diabetes [1].

Over 20 potential therapeutics have been tested in clinical trials in FA patients, mainly in small pilots with a few larger later-stage trials, but no drug has been approved for this disease. Typically, trials have been conducted over less than a year using a clinical end point such as the Friedreich’s Ataxia Rating Scale (FARS) or Scale for Assessment and Rating of Ataxia (SARA) [2,3]. These scales effectively measure disease progression in FA patients and use of a modified version of FARS (termed mFARS) has been accepted by regulators as a potential approval end point. However, power analyses show that trials using these end points need to be conducted over at least 1–2 years to see an effect for a drug that slows or stops disease progression [2,3]. Similar lengths of trials are needed if functional end points such as the 25-foot walk or 9-hole peg test are used [4,5]. While a reversal of disease symptoms is highly desirable, patients also value potential therapeutics that slow progression [6]. Development of such therapies remains challenging as trials that take over a year are expensive and difficult to conduct.

The FA field needs an approach to identify drugs that are likely to be effective based on measurements that can be made more quickly than changes in clinical scales. This will allow prioritization of therapies that are most likely to be effective, so that they can be moved into longer trials where true clinical benefit can be measured. Biomarkers are defined by the US FDA as ‘characteristics that are objectively measured and evaluated as an indicator of normal biological processes, pathogenic processes, or pharmacologic responses to a therapeutic intervention’ [7]. Use of appropriate pharmacodynamic and monitoring biomarkers may offer an opportunity to make quicker evidence-based decisions on potential therapeutics, which will accelerate drug development for the disease and encourage additional companies to investigate potential novel therapies.

Proposed FA biomarkers may assess changes in the affected gene product, frataxin (FXN), or downstream epigenetic, mRNA or protein changes. Gene expression and proteomic profiles can illustrate the broad range of secondary pathways disrupted as a result of the mutation. Metabolic assessments, either targeted or unbiased, detect changes in further downstream events. Structural assessments (imaging and histology) of target tissues provide information on pathological changes in such tissues. Loss of critical tissues then results in functional changes, such as loss of nerve conduction. All of these may be measured as potential biomarkers. Further downstream, these result in derangements such as motor deficits, visual and hearing loss and cardiac failure, which may be measured using clinical outcome assessments.

Considerable effort has been dedicated to the identification of FA biomarkers for use in drug development. Pharmacodynamic biomarkers could offer rapid data supporting whether a potential therapy is hitting its target. Monitoring biomarkers could demonstrate if a potential therapy is slowing progression of disease, or of specific symptoms, giving early evidence of potential clinical effect. Different monitoring biomarkers may be needed in different systems affected by FA, such as the heart and CNS and peripheral nervous system, as not all potential therapies are expected to help all symptoms. Prognostic biomarkers that predict when new symptoms (e.g., cardiomyopathy) become clinically relevant or define groups of patients likely to develop new functional losses may also add value.

The 2018 Friedreich’s Ataxia Biomarker Meeting was held at the Center for Advanced Medical Simulation and Learning in Tampa, FL, USA; with the goal of reviewing the state of biomarker development for FA and determining next steps to develop the biomarkers needed for various contexts of use to accelerate drug development. The meeting also included data on speech as a novel clinical end point. Moderators led discussion around prioritizing markers and around next steps in validating those biomarkers with the greatest chances of success. In this meeting report, we summarize data that were presented on the most promising biomarkers, their proposed contexts of use and conclusions on what is needed to further develop such markers for drug development.

## Analysis of frataxin proteoforms in whole blood & tissue samples

In FA, where there is a deficiency in FXN protein, many pharmacological approaches to increasing FXN levels are being developed [8]. Therefore, development of a sensitive and specific assay to detect active FXN is desirable to measure FXN protein as a monitoring biomarker for FXN-inducing therapies. Treatment of fibroblasts with chemical and genetic modulators have similar effects on FXN levels expression in cells from both healthy controls and FA patients, which express lower amounts of FXN. However, a number of treatments change the total amount of full-length and intermediate forms of FXN with no effect on the levels of mature (active) FXN [9]. Therefore, it is essential to be able to quantify the biologically active mature 14 kDa isoform of FXN (amino acids 81–210) in order to assess the effect of therapeutic interventions designed to increase FXN expression in FA patients. Ian Blair from the University of Pennsylvania presented a novel MS-based assay for measurement of active isoforms of FXN in blood.

Development of a blood-based assay for FXN provides a formidable bioanalytical challenge because in blood the biologically active form of FXN is only present in blood cells and is undetectable in serum or plasma. Originally, it was thought that whole blood could not be used for analyzing mature FXN levels because it was present in long-lived erythrocytes (half-life 115 days), which provided the major source of mature FXN in blood from both controls (average: 70 ng/ml) and FA patients (average: 17 ng/ml) [10]. Measurement of FXN levels in whole blood would not be compatible with shorter therapeutic trials that typically last for less than the half-life of erythrocytes, and so would not reflect the change in mitochondrial FXN levels. To overcome this problem, a dipstick immunoassay approach was developed for the analysis of mitochondrially-derived mature FXN in platelets [10–14,12]. Although the dipstick assay was rapid and convenient it only had a sensitivity of 76.5% and a specificity of 66.7%; FXN values for four of nine controls overlapped with 17 of the FA cases and values from three of the FA cases overlapped with the nine controls [12]. Therefore, a highly specific and sensitive assay for quantifying mature FXN levels in platelets was developed. The assay, based on stable isotope dilution LC–MS, has 100% sensitivity and specificity for discriminating between controls and FA cases [15].

**Figure 1.**
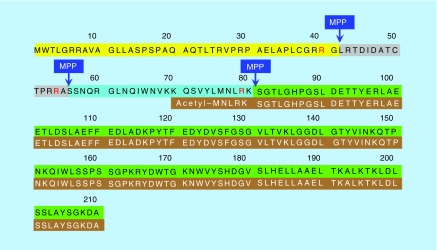
The structure of frataxin protein. Full-length frataxin (1–210, yellow, gray, cyan, green), intermediate form-1 (42–210, gray, cyan, green), intermediate form-2 (55–210, cyan, green), mature frataxin (81–210, green) and isoform E (76–210, brown). MPP: Mitochondrial processing peptidase, which cleaves at the R-2 sites.

Analyzing platelets on a routine basis is challenging in clinical settings. To overcome this issue, Blair showed data demonstrating that the LC–MS approach can readily distinguish a novel proteoform of FXN that is found erythrocytes, termed isoform E. Isoform E can be measured from whole blood samples making sample acquisition applicable to all clinical settings. Isoform E contains 135-amino acids of the full FXN protein (76–210) and an N-terminally acetylated methionine residue (Figure 1) [16], and arises through alternative splicing compared with that used for the canonical full-length form of FXN (Figure 1; 1–210) [16]. There are three-times as much isoform E in erythrocytes (26.7 ± 6.4 ng/ml) from the blood of healthy volunteers (n = 10) when compared with the mature mitochondrial FXN present in other blood cells (7.1 ± 1.0 ng/ml) [16]. The Blair laboratory has recently found (using a stable isotope dilution LC–MS assay) that both isoform E (8.5 ± 1.1 ng/ml) and mature mitochondrial FXN (2.1 ± 1.1 ng/ml) are reduced by more than 70 % in blood from FA patients with homozygous GAA trincleotide repeats (n = 54) when compared with healthy control subjects, suggesting that isoform E levels correlate with other isoforms of FXN, and thus isoform E measurements may reflect total FXN levels and be a useful biomarker in studies of FXN upregulating therapies.

The ability to quantify extramitochondrial and mitochondrial proteoforms of FXN in whole blood using rigorously validated assays will make it possible in the future to monitor the efficacy of therapeutic interventions designed to elevate FXN levels. Furthermore, the assays can be performed in tissue samples, which means that it will also be possible to monitor the levels of both FXN proteoforms in muscle biopsies from FA patients being treated with conventional therapies as well as those being treated with gene therapy and CRISPR-based approaches where blood levels might not reflect the upregulation of FXN expression. This will require validation studies to be conducted in animal models to confirm that muscle can act as a surrogate tissue for brain and heart tissues in primate models such as the cynomolgus monkey (crab-eating macaque). There are only three amino acid differences (D^92^/E^92^, R^168^/W^180^, G^187^/A^187^) between cynomolgus monkey (Uniprot # Q8HXX9) and human (Uniprot # Q16595) mature FXN; none of the antibodies that are currently available can distinguish these two forms. It is unlikely that it will ever be possible to raise antibodies with such constrained specificity and so LC–MS-based methodologies provide the only approach that will be able to distinguish the monkey and human mature FXN in the foreseeable future [15,16]. It will also be essential to show that the correct mature FXN sequence is being generated by therapeutic interventions. In view of the very low tissue concentrations in healthy muscle samples (typically 600 pg/mg tissue), this will require the use of high-sensitivity MS/MS approached such as those described recently for high mobility group box-1 protein [17]. Clearly, bioanalytical approaches for quantification of FXN levels in blood and tissue to assess the efficacy of future pharmacological-, gene therapy- and CRISPR-based therapeutic approaches to treating FA will rely heavily on the use of stable isotope dilution LC–MS-based methodology.

## Epigenetic silencing & methylation of the *FXN* gene

FA is caused by low levels of the protein FXN, which occurs due to the epigenetic silencing of the *FXN* gene as a result of an expanded GAA repeat in the first intron of the gene [18]. One therapeutic approach to increasing *FXN* levels is through reducing this epigenetic silencing and thus increasing *FXN* gene expression [10,19–23]. However, early results with HDAC inhibitors demonstrated that there is a critical balance between acetylation and methylation of histone H3K9 at the *FXN* locus that determines the transcriptional status of the gene [23]. Thus, the effectiveness of HDAC inhibitors in increasing *FXN* expression may be variable dependent on the existing degree of methylation and acetylation of the endogenous *FXN* gene in individual patients. In a Phase I trial of the HDAC inhibitor 109, one out of five patients tested at the highest dose did not show any increase in *FXN*, and was proposed to be a nonresponder [23]. Sanjay Bidichandani from the University of Oklahoma Health Sciences Center discussed work that his group has been doing around the measurement of the degree of DNA hypermethylation of a *FXN* CpG island (CGI) shore, which is an established mechanism of human gene silencing. The *FXN* gene is silenced by increased levels of repressive histone H3K9me3 and H3K27me3 chromatin modifications. This is similar to X chromosome inactivation, wherein H3K9me3 occurs early, followed by H3K27me3, and finally DNA methylation that stabilizes the repressive chromatin. Bidichandani showed that DNA hypermethylation in the CGI shore of *FXN* is dependent on the length of the triplet repeat sequence, occurs in disease-relevant tissues and is widespread in iPSC-derived FA neurons. Lymphoblastoid cell lines from FA patients with atypically low levels of DNA methylation showed higher efficacy of gene reactivation via HDAC inhibition, and chemically induced demethylation potentiated the gene reactivation ability of HDAC inhibition. This suggested that the degree of methylation might be predictive of the ability to reactivate the transcription of the *FXN* gene. Bidichandani presented data from a prospective study of 48 people with FA; testing freshly isolated peripheral blood mononuclear cells for response to HDAC inhibitor treatment and how this corresponded to levels of *FXN* CGI shore methylation. Preliminary results indicated that lower CGI shore methylation correlates with superior response to HDAC inhibitor treatment in FA, suggesting that DNA methylation measured in cells from patient blood may be an effective biomarker for prediction of patients who may respond to HDAC inhibition treatment.

## -Omics approaches to identify new Friedreich ataxia biomarkers

Unbiased high-throughput -omics approaches based on screening of a large number of traits (genes/proteins/metabolites, etc.) may enable identification of biomarker candidates downstream from loss of FXN that may be missed by more targeted approaches. Several groups have conducted -omics analyses in various FA models to identify novel biomarker candidates, two of which presented their unpublished findings at this meeting. Marek Napierala from the University of Alabama at Birmingham presented data from his group, where they used a proteomic screen to investigate potential protein expression changes in primary fibroblast and serum samples obtained from FA patients and unaffected controls. Their approach utilized reverse-phase protein array, a sensitive and high-throughput method allowing acquisition of expression data for 216 unique protein epitopes. A total of 67 fibroblast samples (49 FA and 18 controls) were analyzed and the levels of 30 proteins significantly differed in FA fibroblasts compared with control cells (p < 0.05), signaling molecules and metabolic enzymes predominated in this group. One of the most significantly upregulated proteins identified in the reverse-phase protein array dataset was ALDH1A3. Bioinformatic analyses uncovered a statistically significant correlation between the levels of this enzyme and the presence or absence of cardiomyopathy. As ALDH1A3 cannot be detected in serum or plasma, present efforts focus on quantitation of its metabolites within the retinoic acid pathway in serum samples collected from patients and controls. If successful, levels of the substrates and/or products of the ALDH1A3 enzyme could be further validated as a prognostic or predictive biomarker of cardiomyopathy in FA.

Helene Puccio from IGBMC Strasbourg reported results of -omics analyses conducted primarily using a mouse model of FA. Her group focused on discovering biomarkers associated with the strong cardiac phenotype presented in a well-characterized conditional *Fxn* knockout mouse model (*Mck* cardiac model) [24]), followed by secondary validation in human samples. Transcriptome analyses of mouse heart tissue revealed upregulation of the activation transcription factor 4 (Atf4) network in *Mck Fxn* KO mice. Atf4 is a master regulator of stress response and governs transcription of numerous genes, including Gdf15 and Fgf21. These genes were designated as biomarker candidates by Puccio’s laboratory. In *Fxn* deficient mouse heart tissue, gene and protein expression of both candidates was significantly upregulated, however, these results did not translate when human samples were tested. Further studies in humans using samples from age-matched control and FA cohorts are the focus of ongoing efforts to validate GDF15 and FGF21 as cardiac biomarkers.

Puccio’s group also observed changes in one-carbon metabolism as well as amino acid synthesis and degradation pathways in the hearts of the *Mck* mice. Transcriptome analyses revealed dysregulation of several genes involved in folate/nucleotide metabolism and closely linked amino acid metabolism. Puccio’s group confirmed expression changes identified using integrated omics approaches and subsequently performed specific metabolite analyses using mouse heart tissue, plasma and urine. Significant changes in concentrations of several amino acids, including metabolites of the one-carbon pathway and branched amino acid metabolism were detected in *Mck* mouse heart tissue compared with controls, but not in plasma obtained from these animals. Interestingly, levels of some of the amino acids analyzed in urine differed between these groups indicating that comprehensive analyses of patient urine could lead to identification of novel cardiac/metabolic biomarkers of FA.

These -omics studies highlight an important caveat of current biomarker discovery efforts. Using *in vitro* cultured cells, albeit primary and derived from FA patients or tissues from validated FA animal models, allows for precise control over experimental conditions and standardization of protocols for sample treatment and acquisition. However, substituting these models for patient material (serum, plasma, spinal fluid, muscle biopsy, etc.) carries a high risk of false positive/negative results due to biological variation, dietary, lifestyle or environmental factors. Inevitably, validation of biomarkers discovered in experimentally controlled model systems requires the use of many patient samples to overcome the ‘noise’ – if even possible. Prospectively, it may be useful to consider developing standard operating procedures for collection of samples to be analyzed on omics-based platforms for biomarker discovery that take into account or document certain criteria in order to minimize external influences. The same criteria can later be applied when samples are collected for validation and/or assessment of biomarkers. This approach, although initially burdensome especially considering regulatory and logistic efforts to ensure appropriate standardization of material acquisition, storage and analyses, could eliminate lengthy and frequently unsuccessful validation efforts of biomarker candidates discovered in various experimental *in vitro* or animal models.

## Metabolic biomarkers

FXN functions in a critical process of cellular metabolism, biosynthesis of iron–sulfur (Fe–S) clusters. Numerous proteins, especially enzymes-catalyzing redox reactions during energy production, require Fe–S clusters for proper function. Several studies indicated that metabolic changes can accompany decreased FXN levels. Pierre-Gilles Henry’s group from the University of Minnesota presented results of an exploratory study aimed to measure the rate of the Krebs cycle (or tricarboxylic acid [TCA] cycle) directly in FA dentate nucleus, a structure with confirmed pathology in FA [25]. His group utilized ^13^C magnetic resonance spectroscopy (MRS) following administration of a ^13^C-labeled glucose substrate and monitored its conversion to the ^13^C-labeled amino acid glutamate. As glutamate can be produced by transamination of 2-oxoglutarate (TCA intermediate), isotope-labeled carbons from glucose can be detected in this amino acid and the kinetics of the ^13^Cincorporation into glutamate reflects the rate of the TCA cycle. Rather unexpectedly, analysis of six FA and six matched control subjects revealed a higher rate of the TCA cycle in dentate nucleus in the patient cohort, perhaps reflecting a compensatory effect caused by progressive atrophy of the dentate. Further studies in a larger group, including FA patients with different disease stages, should reveal the potential applicability of *in vivo* TCA cycle analyses as an FA biomarker.

A different approach to noninvasive assessments of metabolic changes in FA patients was presented by Shana McCormack’s laboratory from the Children’s Hospital of Philadelphia. Results from two entirely different metabolism analyses were discussed. First, a creatine chemical exchange saturation transfer (CrCEST) technique was used to detect mitochondrial oxidative phosphorylation capacity *in vivo*, in lateral and medial gastrocnemius and soleus skeletal muscle groups in the calf. The CrCEST technique allows for muscle-group-specific determination of free creatine levels. When used in combination with in-magnet exercise, CrCEST signal indirectly reflects OXPHOS capacity of the muscle with high anatomic precision. The proof-of-concept of this approach was established earlier by this group through analysis of small cohorts of patients suffering from various mitochondrial diseases [26]. The presented work, conducted in both adults and children/adolescents with FA and control groups, demonstrated a prolonged postexercise CrCEST recovery in FA patients indicating that CrCEST could be an appropriate *in vivo* longitudinal biomarker reflecting OXPHOS muscle capacity. Extension of this work into cardiac muscle as well as longitudinal analyses is certainly warranted.

The second part of the presentation was focused on glucose metabolism. Aberrant glucose handling is tightly connected to FA and mechanistically linked to deficits in mitochondrial function. Although only approximately 10% of FA patients suffer from diabetes mellitus, a significant majority of adults with FA demonstrate abnormal glucose homeostasis, which may respond to therapy and hence could serve as a surrogate biomarker. In fact, assessment of glucose metabolism was used as an outcome measure in a clinical trial of the α-tocopheryl quinone, EPI-A0001 [27]. In the current study, the McCormack group utilized stable isotope tracers (1-^13^C-glucose) to conduct oral glucose tolerance tests in FA and control cohorts with monitored BMI. The results confirmed that OGTT is clinically relevant and may represent an important outcome measure, especially with improved methodology, such as including a stable isotope glucose tracer to account for enteral absorption. The nondiabetic FA patient cohort demonstrated a higher fasting level of glucose compared with the control group. In addition, patients showed a higher production of insulin (postprandial) that may be necessary to achieve similar levels of glucose as control individuals. Finally, nondiabetic individuals with FA had prolonged elevation of lactate levels after the oral glucose load as compared with controls, thus lactate metabolism during the OGTT could also represent a metabolic biomarker in FA.

## Neuroimaging approaches to Friedreich ataxia biomarkers

Neuroimaging can be a crucial resource to assess structural integrity and metabolic features of the CNS, and several groups are using such techniques to try to identify FA biomarkers. Pierre-Gilles Henry and Christophe Lenglet from the University of Minnesota, Marcondes Franca from University of Campinas, Brazil, and Ian Harding from Monash University in Melbourne, Australia, reported their findings using several neuroimaging approaches. The Minnesota group used structural imaging to show that spinal cord morphology at cervical 2–3 level was abnormal in FA subjects, with lower cross-sectional area and higher eccentricity. MRS showed that total N-acetylaspartate (tNAA) was decreased and myo-inositol (mIns) was elevated in cervical cord in FA, resulting in decreased tNAA/mIns ratio. Both the morphological and biochemical measures showed significant longitudinal changes over 12–24 months. Interestingly, observed tNAA/mIns values in early-stage FA patients suggest that values may have started to decline well before symptom onset, perhaps due to incomplete myelination during development. The group also presented data on diffusion tractography studies, an imaging technique that creates images of the white matter in the brain using anisotropic diffusion. Fiber tractography is a 3D reconstruction technique to access neural tracts using data collected by diffusion tensor imaging (DTI). At the cervical level 4–5 in the spinal cord, fractional anisotropy (which reflects fiber density, axonal diameter and myelination in white matter) was significantly lower in FA than in controls, and declined longitudinally over 36 months of follow-up. This was mirrored by an increase in mean diffusivity – a measure of the movement of water that reflects brain structural integrity. Several MRI measurements correlated with disease severity measures. Significant changes in fractional anisotropy and radial diffusivity values were also noted in several brain regions, especially the cerebellar peduncles and corticospinal tract. Franca’s presentation documented similar findings on brain DTI in Brazilian FA patients, and also identified changes in the corpus callosum. In the Brazilian cohort, spinal cord area declined and eccentricity increased. Harding provided imaging data showing increasing dentate iron and loss of dentate volume over 2 years of follow-up. He also documented loss of volume in several cerebellar lobules and changes in fractional anisotropy in cerebellar white matter. Longitudinal changes tended to be more easily discernible in early stage patients.

In addition to the data presented in this meeting, there is additional literature, mostly cross-sectional, on an array of abnormalities in various cerebellar and cerebral structures in FA that may be documented with imaging techniques [28]. However, longitudinal data are still relatively scarce. It may be necessary to identify key features that look most promising for further analyses and replication in larger cohorts and across multiple sites. In terms of structural imaging, spinal cord atrophy and DTI measures in cerebellar peduncles and corticospinal tract seem to stand out as having been consistently documented in multiple studies and at multiple sites. However, many of these structural changes still take up to a year to show significant changes with disease progression, depending on the exact measure and the stage of disease. Further research into rapidly progressing populations and improvements in MR techniques may improve the sensitivity of such markers. Nonstructural imaging, such as MRS and iron imaging may give more rapid measures of drug response.

## Structural & functional biomarkers of the nervous system

The structural integrity of the nervous system can also be assessed with markers of neural injury and repair that may be measured in body fluids. Kristin Obrochta (BioMarin Pharmaceutical, Inc.) discussed the value of serum neurofilament light chain (NFL) in FA. Neurofilaments are abundant structural proteins exclusively present in neurons and levels of their components tend to increase in body fluids as a result of axonal damage in a variety of circumstances [29]. Recent methodologies such as the single-molecule array technology reported in this presentation improve sensitivity of NFL detection to allow for more reliable measurements, including in blood samples. The data presented showed that NFL levels in serum were higher in samples from 35 FA subjects compared with controls. Repeat samples collected up to 24 months later showed minimal directional trends, with most longitudinal values falling within 30% of the baseline values. NFL levels correlated negatively with age at onset and with age at sampling, which are confounded variables in samples collected near the time of diagnosis in the FA population. Paola Giunti’s team from University College London, reported similar results in a poster presentation, showing increased NFL levels in a different cohort of FA patients, as well as increased levels of glial fibrillary acidic protein and ubiquitin C-terminal hydrolase L1, also markers of neurodegeneration [30].

This type of quantifiable molecular marker that reads out affected tissue pathology from blood is attractive because of the possibility for use in large multicenter cohorts and centralized analyses and if validated, will be less expensive than imaging. In addition to FA, NFL levels in CSF and blood are clearly distinguished between patients and controls in several neurodegenerative diseases, but corresponding longitudinal data are still scarce [29].

Loss of large-myelinated sensory fibers is well recognized in FA, with limited data also suggesting loss of small-unmyelinated fibers [31–33]. Peter Creigh from the University of Rochester showed data from *in vivo* reflectance confocal microscopy measures that quantitate Meissner’s corpuscles (MC), the main touch pressure sensory receptors in hands and feet, and from epidermal nerve fiber density (ENFD) measures via skin biopsies, as well as studies of quantitative sensory thresholds in FA and control subjects. All procedures were well tolerated except for the skin biopsies in children. MC densities were significantly diminished in FA in all regions measured (digit 5 of the hand, thenar eminence and arch of foot). Quantitative sensory functional measures including touch pressure, vibration and cooling thresholds were also significantly impaired in FA. Significant reductions in ENFDs were only detected in more severely affect patients, although ENFD analyses were limited by smaller group sizes. There were moderate-to-strong correlations between MC densities, ENFDs and most of the functional sensory testing measures with more global disease severity assessments. Significant worsening of the measures occurred over 6–12 months on longitudinal analysis. These are innovative methods for quantitating sensory dysfunction in FA, published previously in other neuropathies (e.g., [30]). Similar methodology has been used to quantitate unmyelinated nerve fibers in the cornea as well and may also have utility in FA, although this was not discussed at the most recent biomarker meeting [35].

Massimo Pandolfo (Université Libre de Bruxelles, Hôpital Erasme) presented data on the use of proprioceptive assessments using magnetoencephalography. While proprioceptive loss is already severe at symptom onset and thus may not show further measurable changes, this may reflect the insensitivity of measures of the proprioception and the limited components of the proprioceptive system that are routinely examined. The Brussels group measured corticokinematic coherence (CKC) that reflects movement-related somatosensory inputs to the SM1 cortex. The measure had good reproducibility and both the latency and amplitude of the responses differed significantly from controls. The CKC values correlated with GAA repeat size indicating some biological validity, and that the values reflect genetic severity more than clinical status. There was low sensitivity to progression over 1 year. Other magnetoencephalography-related measures that were explored included resting state functional connectivity and somatosensory mismatch negativity, which were abnormal in FA. There are other methods for measuring proprioception [32,33] that are based on quantifying traditional methods of examination, such methods have not been explored in FA. In an unpublished study of a directed movement using foot extensors in FA, it has been found that subjects make erroneous estimates of a movement they have just performed. Additional studies are needed to establish that continued decline of proprioception occurs in FA subjects after disease onset and this is measurable.

Several of the reported neurological measures show promise as monitoring biomarkers. Further longitudinal assessment in larger patient cohorts is needed in order to fully understand the sensitivity and rate of change of such measures, but data so far suggest that measures such as MC density, quantitative sensory testing and proprioception assessed by CKC may have potential to determine changes in neurological function over the course of disease. Current studies have only been performed in small cohorts at single sites, so additional work is required to determine whether such measures can be reliably assessed at multiple sites and with multiple operators, as well as to demonstrate robust changes that might be seen in the course of a clinical trial. In contrast, quantitation of NFL has been clearly demonstrated in other neurological diseases to be achievable at multiple sites in a robust manner. Further work is required in FA to understand how NFL levels change with disease, and how they may change with potential treatments.

## Cardiac imaging biomarkers

Heart malfunction is the primary cause of early mortality in FA: mean life expectancy is reduced to approximately 40 years, and approximately 60% of the patients with FA will suffer fatal cardiac complications, most commonly heart failure, or complications associated with heart failure such as stroke secondary to atrial fibrillation [34,35]. Biomarkers reporting on cardiac involvement in FA are needed for three reasons: early identification of those patients who are at risk of developing fatal cardiomyopathy, identification of those patients needing therapeutic cardiac intervention at an earlier stage in their disease course when such therapies become available and identification of biomarkers reflective of disease pathobiology that can be used as surrogate markers of outcome. Six presentations at the meeting addressed the topic of cardiac biomarkers, addressing both imaging technologies and potential serum biomarkers reflective of cardiac involvement.

Of those imaging technologies currently employed for cardiac evaluation, the use of MRI holds the most promise in FA. Kimberly Lin (Children’s Hospital of Philadelphia) reviewed the application of cardiac MRI (cMRI) to cardiomyopathies in general, and FA specifically. cMRI is feasible in most FA patients and is widely available for standard imaging protocols including volumetric analysis, functional quantification and myocardial strain. Inter- and intra-observer variability is excellent for accurate assessment of biventricular volumes, mass and function [36]. It is considered as the ‘gold standard’ when compared against echocardiography because of its quantitative capabilities and unparalleled image acquisition. Echocardiography, on the other hand, is relatively fast and well tolerated, does not suffer from interference by surgical hardware or metal, and is readily available across virtually all medical centers with standardized views and assessments. Like cMRI, it provides data on cardiac function, structure and volume, and strain.

Where cMRI may provide significant advances in FA is in its ability to interrogate tissue metabolism and characteristics. Several cMRI approaches were discussed. Late gadolinium enhancement can be used to identify myocardial fibrosis. In patients with nonischemic cardiomyopathy, it is predictive of adverse cardiovascular events [37] and has been applied to the FA heart as a combinatorial tool with other biomarkers [38]. However, its predictive capability as an independent tool for the FA heart has not yet been determined, and late gadolinium enhancement may be sensitive only in the advanced stages of cardiomyopathy. Further work is urgently needed in this area. Myocardial deformation as quantified by cMRI may be able to detect very early abnormalities in cardiac pathology for FA and can provide excellent long-term follow-up. Key obstacles in its use for clinical and research purposes include discrepancies in results across the various software platforms, and lack of studies evaluating long-term outcome relative to cMRI strain analysis [39]. Myocardial perfusion reserve (MPR) has been evaluated in FA, has been published and is informative [40]. In this technique, the perfusion of the myocardium at rest and with stress during a cMRI is determined with administration of adenosine, a vasodilator, in conjunction with an imaging agent such as gadolinium. Abnormalities in MPR reflect defects in micro- and macro-circulatory reserve and correlate with serologic findings of metabolic syndrome in FA. This technique has been associated with cardiovascular risk factors in obese patients with heart disease [41]. Whether changes in MPR can independently predict outcome or clinical course in FA remains to be determined. In particular, it is unknown if MPR can serve as a dynamic imaging biomarker reflecting timely improvement in cardiovascular microcirculation with administration of a therapeutic agent, and if it adds additional predictive capability that traditional markers of metabolic disruption, such as serum lipid profile and insulin levels, do not already provide. Cardiac MRS can be used to estimate the energetic state of a tissue, such as heart. Lodi *et al.* reported that cardiac bioenergetics were abnormal in FA patients in the absence of any deterioration in contractile performance [42]. They showed the phosphocreatine to ATP ratio (PCr:ATP) was abnormal in the FA heart, with or without hypertrophy, when compared with controls. The rate of ATP flux through creatine kinase has recently been shown to predict heart failure outcomes in non-ischemiccardiomyopathy via cardiac MRS [43]. This is an exciting biomarker because it reflects metabolic changes early in the FA heart and may be responsive to therapeutic interventions. Limitations of this technology currently include lack of generalized availability. Finally, the role of T1 mapping by cMRI in FA was discussed. This has been proposed by multiple investigators as a noninvasive alternative to cardiac biopsy for tissue characterization. T1 mapping may inform on underlying disease processes such as fibrosis and edema. A key feature of T1 mapping is its quantitative output, which is attractive for longitudinal studies in clinical trials and is independent of cardiac remodeling and hypertrophy. This technique can quantify small variations in cardiac tissue and can quantify diffuse fibrosis in cardiac tissue since it does not depend on contrasting intensity. It is unknown, however, if T1 mapping will be able to detect subtle differences in cardiac fibrosis in the setting of FA. A study between Melbourne and Children’s Hospital of Philadelphia is ongoing and will determine the feasibility of conducting cMRI studies, such as T1 mapping, across institutions in the disease of FA. Preliminary data in 30 patients did not show significant change over the course of 1 year.

Marcondes Franca (University of Campinas, Brazil) presented imaging data looking at left ventricular myocardial remodeling in patients with FA. In this study, they examined 26 FA subjects, and 10 controls by cMRI T1 mapping to determine the state of myocardial interstitial fibrosis and cardiomyocyte hypertrophy, termed Tau or τ_ic_, and then correlated this with the FARS scores determined at that time. Use of cMRI to quantify cardiomyocyte size in remodeling was described by Coelho-Filho *et al.* in 2013 [44], but had not previously been applied to the mitochondrial disease of FA. Not surprisingly, they found that FA patients had predominantly normal LV ejection fraction with significantly increased left ventricle (LV) mass index (LVMASSi) and decreased LV end-diastolic volume index when compared with healthy controls. As a result of this remodeling, the heart rates of the FA subjects were significantly higher than controls. What was exciting, however, was that the found both the extracellular volume (ECV) and cardiomyocyte fiber size (τ_ic_) were significantly increased in the FA patients when compared with controls. Furthermore, the FARS scores from these FA subjects correlated negatively with cardiomyocyte size, τ_ic_. Both the LVMASSi and the cardiomyocyte mass index ([1−ECV] × LVMASSi) correlated negatively with age, indicating that LV hypertrophy may transition at later age to a more ominous form of cardiomyopathy, such as a dilated or restrictive cardiomyopathy. Thus, in this early study of FA patients studied by cMRI, LV hypertrophy and interstitial expansion were found to be hallmarks of an early progressive cardiac phenotype but were not associated with the neurological decline that occurs with more advanced age. However, Franca *et al.* have demonstrated a correlation between the cardiomyopathy and neurologic phenotype in FA by showing that the cardiomyocyte size was associated with the FARS neurological score independent of age and ECV. Given that previous investigators have demonstrated that cardiomyocytes in FA are hypertrophied and this is due to expansion of the mitochondrial pool within the cardiomyocyte [25], it is exciting to speculate that cardiomyocyte hypertrophy as measured by Tau could be a quantitative and rapid pharmacodynamic biomarker. Whether Tau could be predictive of a change in neurologic outcome as well remains to be seen and needs further study in a larger cohort.

## Cardiac biomarkers: integrated approaches

Several investigators took an integrated approach to quantifying cardiac disease in FA using multiple modalities such as serum biomarkers, exercise and cMRI. One such study was presented by Kim Lin (Children’s Hospital of Philadelphia) and examined a serum biomarker panel as correlated with cMRI. This was a multi-institutional study between Children’s Hospital of Philadelphia and Murdoch Children’s Research Institute in Australia. They examined the hypotheses that serum markers of cardiac failure, fibrosis, injury and inflammation would be higher in FA subjects than non-FA controls, and that these markers would correlate with cardiac MRI-derived markers of disease severity. To do this, they used a multiplex platform to analyze multiple serum biomarkers that reflect heart failure (NT-pro BNP, ST2), fibrosis (galectin-3, PIIINP), injury (hsTnI, FABP-3) and inflammation (MCP-1 and highly sensitive C-reactive protein) in both FA (n = 98) and control (n = 58) subjects. Cardiac phenotype was assessed by a standard cMRI protocol that included volumetric data as well as T1 mapping and calculation of ECV.

They found that PIIINP was higher in FA versus non-FA subjects but there were no other biomarker differences between cases and controls. Among the FA subjects, PIIINP showed a positive correlation with cMRI measures of remodeling and fibrosis, including left ventricular mass volume ratio, LV mass index and ECV. NT-pro BNP was also correlated with ECV but not with LV mass index or LV mass volume ratio. No other biomarker correlations were seen. Thus, PIIINP, a marker of fibrosis, shows promise as a marker of cardiac involvement in FA but its significance will require longer-term follow-up to determine its relationship to outcome.

In a similar, multimodality approach, investigators from the University of Florida applied cMRI, echocardiography and metabolic exercise testing to 26 FA and 16 control subjects. The goal of this project is to enroll 50 FA subjects and follow them longitudinally over 5 years with a testing interval of 6 months. This study will be used to identify outcome measures that could be applied to the development of future clinical therapeutic trials in FA. They also assessed fibrosis using a novel strategy called Cine-Balanced Steady State Free Precession, which does not require contrast during imaging. Their preliminary analysis showed signs of fibrosis, even in asymptomatic patients. A key finding of this study could include the correlation between early fibrosis in the heart, such as at the time of FA diagnosis, and cardiac outcome over time.

Kristin Obrochta (BioMarin Pharmaceutical, Inc.) presented novel findings on cardiac troponin (cTnI), which is widely used as a marker of cardiac damage during heart attacks. In 35 FA subjects, they found cTnI to be independently elevated in a subset of FA subjects. Long-term outcome studies, and correlation with other modalities, such as cMRI and FARS scores, will provide context for the use of these biomarkers that are readily accessed from blood samples.

In summary, certain cMRI and serologic measures show promise, and should be validated in larger populations across multiple sites. Integration of multiple modalities will be crucial. This is because the utility of certain isolated biomarkers, such as cTnI, are currently unknown for FA and may provide better prediction of outcome when used in a panel rather than a single biomarker. Thus, longitudinal studies using novel assays compared with validated neurologic or cardiac assays, such as the FARS score, will help determine their usefulness in this disease. There is considerable value in looking at cardiac biomarkers that are observed to change in other forms of heart disease and comparison of these biomarkers to FA clinical outcome data is vital.

## Novel outcome measures for Friedreich ataxia

Adam Vogel (Melbourne School of Health Sciences) presented information on speech analysis. Speech issues include the basic qualities of speech, intelligibility to others, quality of life changes because of dysarthria and relationship to other disease measures. The study objectively analyzed qualities of dysphonia and motor speech from sustained vowel and connected speech from 117 patients. Various dysphonia measures correlated significantly with FARS with values of 0.37–0.41. Timing measures such as speech rate, duration, percent pauses obtained from paragraph reading also had significant correlations with FARS with values of 0.49–0.59. Correlations with a quality of life measure (Friedreich's Ataxia Rating Scale) were also significant but values were lower (0.20–0.22 for dysphonia and 0.26–0.30 for timing). No data were provided for reproducibility or longitudinal change. In previous reports, this group has shown that several quantitative features of voice production and speech are abnormal in FA compared with controls but this is also true for a more subjective Auditory Perceptual rating scale [45]. Analyses of speech indicated that certain measures such as utterance duration, rate of spectral change and degree of spectral change declined significantly during follow-up but other measures such as amplitude of rhythm spectra, pause durations – among others, did not change [46]. Speech is a complicated process and ataxic dysarthria is likely amenable to several types of measurements such as speech rate, pause durations, precision of consonants – among others. One of the constraints in this regard is the variability that can be induced by the particular task involved.

The feasibility and validity of clinic-based instrumented measures of balance, gait and dysarthria and dysphonia are being established. The psychometric properties of these methodologies and their sensitiveness to change remain in question. Given the proliferation of such methodologies, it may be prudent for FA researchers to carefully choose selected methods to explore in the larger context of the ongoing natural history studies. The methodology chosen must have acceptable properties in terms of costs, ease of harmonization among many centers and ease of data analysis and also the possibility of detecting trackable presymptomatic abnormalities. Also, the use of home-based wearable devices should be explored in a similar fashion.

## Conclusion

**Figure 2.**
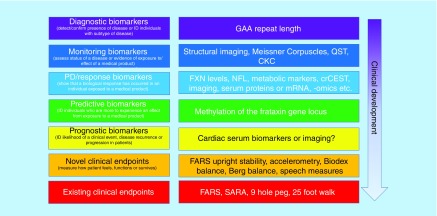
Role of biomarkers in the development of therapies for Friedreich’s ataxia. Biomarkers are in development for many different contexts of use in FA. CKC: Corticokinematic coherence; crCEST: Creatine chemical exchange saturation transfer; FARS: Friedreich’s Ataxia Rating Scale; FXN: Frataxin; NFL: Neurofilament light; mRNA: Messenger ribonucleic acid; QST: Quantitative sensory testing; SARA: Scale for assessment and rating of ataxia.

Considerable progress has been made in the development of biomarkers for FA for various contexts of use (Figure 2). Importantly, new and sensitive assays are now available that can precisely and specifically measure mature-form FXN levels in whole blood in a manner that may be used as a pharmacodynamic or response biomarker for the development of FXN upregulating drugs. Progress has also been made in the understanding of epigenetic differences between patients, which may allow prediction of responders and nonresponders to specific epigenetic-based therapies. Larger datasets and additional data are still needed to further support these biomarkers.

Preliminary data suggest that structural changes in the spinal cord and brain may occur with disease progression and slowing of these changes might reflect slowing of disease. Measures of spinal cord area and DTI measures in cerebellar peduncles and corticospinal tract may provide useful monitoring biomarkers demonstrating the rate of disease progression, although changes with disease course are slower than may be ideal. In the peripheral nervous system, reduction in MC size and density, CKC and quantitative sensory testing also appear to correlate with disease progression and early data suggest that they may show robust changes in studies of 6 months or a year, particularly in younger patients. In each case, changes with disease progression have been demonstrated in relatively small cohorts of patients, and additional longitudinal data are needed to truly demonstrate their sensitivity, specificity and rate of change with disease progression, and multicenter studies are needed to demonstrate the continuity in the measures when recorded at different sites. However, if early data are supported in longer and larger studies, these may provide early insight into whether a proposed therapy may slow disease progression in trials.

A number of biomarkers have been proposed that may be of value as pharmacodynamic or response biomarkers, depending the nature of the therapy in development. Normalization of metabolic changes in FA patients, such as recovery from exercise, glucose tolerance and/or changes in gene expression or protein or metabolite changes may give early indication of a treatment effect of certain drugs. Data are being collected around several of such measures, although demonstration of effect is challenging in the absence of effective therapies for the disease. Where possible, testing of such biomarkers in mouse models of disease where disease can be reversed by expression of FXN might support their use. Reduction in levels of NFL has been proposed as a response biomarker for therapies in several neurodegenerative diseases, and preliminary data suggest that it may also be of value in FA, as levels of NFL are demonstrably high in FA patients.

Biomarkers that reflect FA cardiac disease are still limited in FA, but work is progressing. Several researchers are evaluating cardiac MRI measures and at serum biomarkers to look for prognostic biomarkers that might predict that patients are likely to develop clinical cardiac disease as well as biomarkers for monitoring cardiac function. In neither case have measures been identified that have strong correlation with clinical symptoms at this time. However, the importance of integration of multiple modalities is becoming apparent. This is because the utility of certain isolated biomarkers, in particular fluid biomarkers such as cTnI, NT-pro BNP and PIIINP are currently unknown for FA and may provide better prediction of outcome when used in a panel rather than a single biomarker. Further longitudinal studies using novel assays compared with validated neurologic or cardiac assays in FA populations will determine their usefulness in this disease. Furthermore, more detailed exploration of novel biomarkers, such as cardiomyocyte fiber size, is key to identifying new assays that may report quickly on therapeutic interventions. Again, validation of these biomarkers against clinical outcome data is vital.

Several novel clinical end points have also been proposed to measure FA disease progression. Measures that look at patient speech show promise as clinical end points that could be used in clinical trials. These functions are of importance to patients, and slowing progression of these symptoms is valued, so further understanding of their rate of change, correlation to better established clinical measures such as FARS and sensitivity warrant further investigation. In this meeting, discussion of a few early-stage end points was included, but for the purpose of this report only the section on speech has been included.

Overall, understanding of the types of biomarkers and end points needed for FA is improving, and data have been generated that supports the use of various biomarkers for many different contexts of use in drug development. However, there are still limited data on any marker that robustly shows change with disease progression in less than 6 months, which may reflect the rare of progression of the disease. Disease progression has been shown to be more rapid in patients with early onset and higher numbers of GAA repeats, as well as in younger patients [2], and it may be possible to demonstrate robust biomarker changes more rapidly in these subpopulations. Studies are ongoing to evaluate this. Cardiac biomarkers remain a great unmet need in FA, and further work is required in this area. Development of robust biomarkers for FA remains both a challenge and an opportunity in FA research.

## Future perspective

Biomarker development for FA is progressing rapidly on several fronts. Several possible monitoring biomarkers show promise, and further investigation into such biomarkers (e.g., imaging techniques, measurement of MC) is warranted. Longitudinal studies with more patients will be required to ascertain the rate of change of these markers and their association with disease progression. Pharmacodynamic or response biomarkers, such as metabolic measures and exercise tests also require further testing in larger studies. However, in these cases, it is also important to determine if the measures show correction in the presence of a therapeutic compound. Given the lack of proven therapy for FA, it may be necessary to study such biomarkers in mouse models of disease, where FXN expression can be reduced and restored. Development of cardiac biomarkers are a clear need for the field. Overall, many potential biomarkers have been identified for different contexts of use in FA drug development, and further work will aid us in accelerating drug development for this devastating disease. In the next 10 years, we anticipate many additional therapies reaching the clinic for FA, and the use of these biomarkers will enable faster trials that will give informative answers as to whether to continue development of the therapy. It is an exciting time for drug development in FA.

**Executive summary**

- This paper summarizes the conclusions from the FARA biomarker meeting, on the current state of development of biomarkers for Friedreich ataxia (FA). The conclusions of the meeting were:
  – A new stable isotope dilution LC–MS-based assay to quantitate mature frataxin (FXN) protein is more sensitive and more specific than existing assays and can be used in tissues and whole blood.
  – Preliminary data suggest that the degree of methylation of the FXN locus may be able to predict the efficacy of histone deacetylase inhibitors in upregulating FXN.
  – Metabolic changes, in particular measurement of mitochondrial oxidative phosphorylation capacity in muscle, are promising pharmacodynamic biomarkers; changes in blood glucose may also be a quick, noninvasive biomarker showing normalization of metabolism. Metabolic changes measured by magnetic resonance imaging also show promise as monitoring and/or pharmacodynamic biomarkers.
  – Neuroimaging techniques offer the potential to measure the atrophy of different central nervous system structures that are affected at different disease stages. Larger longitudinal studies are needed to ascertain which structures change in which populations and how rapid such changes are.
  – Levels of neurofilament light chain in blood may indicate axonal damage, and reduction in levels may be a useful pharmacodynamic biomarker.
  – Measures of sensory dysfunction such as the size and number of Meissner corpuscles and epidermal nerve fiber density may be useful monitoring biomarkers.
  – Data on several potential cardiac biomarkers have been collected (metabolic and structural imaging of the heart; fluid biomarkers). Further work is needed to confirm the utility of such measurements for specific contexts of use.

## References

[1] Delatycki MB, Wiliamson R, Forrest SM Friedreich ataxia: an overview. J. Med. Genet. 37(1), 1–8 (2000). 10633128 10.1136/jmg.37.1.1 PMC1734457

[2] Patel M, Isaacs CJ, Seyer L Progression of Friedreich ataxia: quantitative characterization over 5years. Ann. Clin. Transl. Neurol. 3(9), 684–694 (2016). 27648458 10.1002/acn3.332 PMC5018581

[3] Reetz K, Dogan I, Hohenfeld C Nonataxia symptoms in Friedreich ataxia: report from the registry of the European Friedreich’s Ataxia Consortium for Translational Studies (EFACTS). Neurology 91(10), 917–930 (2018). 10.1212/WNL.0000000000006121 30097477

[4] Tai G, Yiu EM, Delatycki MB, Corben LA How does performance of the Friedreich ataxia functional composite compare to rating scales? J. Neurol. 264(8), 1768–1776 (2017). 28695363 10.1007/s00415-017-8566-0

[5] Friedman LS, Farmer JM, Perlman S Measuring the rate of progression in Friedreich ataxia: implications for clinical trial design. Mov. Disord. 25(4), 426–432 (2010). 20063431 10.1002/mds.22912 PMC2954653

[6] Cure FA Foundation. The voice of the patient – Friedrich's ataxia (2017) http://curefa.org/pdf/news/FA-Voice-of-the-Patient.pdf

[7] FDA–NIH Biomarker Working Group. EST (Biomarkers, EndpointS, and other Tools) Resource (2016) www.ncbi.nlm.nih.gov/books/NBK326791/27010052

[8] Strawser C, Schadt K, Hauser L Pharmacological therapeutics in Friedreich ataxia: the present state. Expert. Rev. Neurother. 17(9), 895–907 (2017). 28724340 10.1080/14737175.2017.1356721

[9] Nabhan JF, Gooch RL, Piatnitski Chekler EL, Pierce B, Bulawa CE Perturbation of cellular proteostasis networks identifies pathways that modulate precursor and intermediate but not mature levels of frataxin. Sci. Rep. 5, 18251 (2015). 10.1038/srep18251 PMC4680912 26671574

[10] Plasterer HL, Deutsch EC, Belmonte M, Egan E, Lynch DR, Rusche JR Development of frataxin gene expression measures for the evaluation of experimental treatments in Friedreich’s ataxia. PLoS ONE 8(5), e63958 (2013). 10.1371/journal.pone.0063958 PMC3656936 23691127

[11] Deutsch EC, Santani AB, Perlman SL A rapid, noninvasive immunoassay for frataxin: utility in assessment of Friedreich ataxia. Mol. Genet. Metab. 101(2–3), 238–245 (2010). 20675166 10.1016/j.ymgme.2010.07.001 PMC2996613

[12] Selak MA, Lyver E, Micklow E Blood cells from Friedreich ataxia patients harbor frataxin deficiency without a loss of mitochondrial function. Mitochondrion 11(2), 342–350 (2011). 21147271 10.1016/j.mito.2010.12.003 PMC4419809

[13] Deutsch EC, Oglesbee D, Greeley NR, Lynch DR Usefulness of frataxin immunoassays for the diagnosis of Friedreich ataxia. J. Neurol. Neurosurg. Psychiatry. 85(9), 994–1002 (2014). 24463479 10.1136/jnnp-2013-306788

[14] Lazaropoulos M, Dong Y, Clark E Frataxin levels in peripheral tissue in Friedreich ataxia. Ann. Clin. Transl. Neurol. 2(8), 831–842 (2015). 26339677 10.1002/acn3.225 PMC4554444

[15] Guo L, Wang Q, Weng L Liquid chromatography-high resolution mass spectrometry analysis of platelet frataxin as a protein biomarker for the rare disease Friedreich’s ataxia. Anal. Chem. 90(3), 2216–2223 (2018). 29272104 10.1021/acs.analchem.7b04590 PMC5817373

[16] Guo L, Wang Q, Weng L Characterization of a new N-terminally acetylated extra-mitochondrial isoform of frataxin in human erythrocytes. Sci. Rep.19; 8(1), 17043 (2018). 10.1038/s41598-018-35346-y PMC6242848 30451920

[17] Weng L, Guo L, Vachani A, Mesaros C, Blair IA Quantification of serum high mobility group box 1 by liquid chromatography/high-resolution mass spectrometry: implications for its role in immunity, inflammation, and cancer. Anal. Chem. 90(12), 7552–7560 (2018). 29791130 10.1021/acs.analchem.8b01175 PMC6417096

[18] Campuzano V, Montermini L, Molto MD Friedreich’s ataxia: autosomal recessive disease caused by an intronic GAA triplet repeat expansion. Science 271(14), 1423–1427 (1996). 8596916 10.1126/science.271.5254.1423

[19] Herman D, Jenssen K, Burnett R, Soragni E, Perlman SL, Gottesfeld JM Histone deacetylase inhibitors reverse gene silencing in Friedreich’s ataxia. Nat. Chem. Biol. 2(10), 551–558 (2006). 16921367 10.1038/nchembio815

[20] Lufino MM, Silva AM, Nemeth AH, Alegre-Abarrategui J, Russell AJ, Wade-Martins R A GAA repeat expansion reporter model of Friedreich’s ataxia recapitulates the genomic context and allows rapid screening of therapeutic compounds. Hum. Mol. Genet. 22(25), 5173–5187 (2013). 23943791 10.1093/hmg/ddt370 PMC3842177

[21] Sahdeo S, Scott BD, McMackin MZ Dyclonine rescues frataxin deficiency in animal models and buccal cells of patients with Friedreich’s ataxia. Hum. Mol. Genet. 23(25), 6848–6862 (2014). 25113747 10.1093/hmg/ddu408 PMC4245046

[22] Libri V, Yandim C, Athanasopoulos S Epigenetic and neurological effects and safety of high-dose nicotinamide in patients with Friedreich’s ataxia: an exploratory, open-label dose-escalation study. Lancet 384(9942), 504–513 (2014). 24794816 10.1016/S0140-6736(14)60382-2

[23] Soragni E, Miao W, Iudicello M Epigenetic therapy for Friedreich ataxia. Ann. Neurol. 76(4), 489–508 (2014). 25159818 10.1002/ana.24260 PMC4361037

[24] Perdomini M, Belbellaa B, Monassier L Prevention and reversal of severe mitochondrial cardiomyopathy by gene therapy in a mouse model of Friedreich’s ataxia. Nat Med. 20(5), 542–547 (2014). 24705334 10.1038/nm.3510

[25] Koeppen AH, Ramirez RL, Becker AB The pathogenesis of cardiomyopathy in Friedreich ataxia. PLoS ONE 10(3), e0116396 (2015). 25738292 10.1371/journal.pone.0116396 PMC4349588

[26] DeBrosse C, Nanga RP, Wilson N Muscle oxidative phosphorylation quantitation using creatine chemical exchange saturation transfer (CrCEST) MRI in mitochondrial disorders. JCI Insight. 1(18), e88207 (2016). 27812541 10.1172/jci.insight.88207 PMC5085612

[27] Lynch DR, Willi SM, Wilson RB A0001 in Friedreich ataxia: biochemical characterization and effects in a clinical trial. Mov. Disord. 27(8), 1026–1033 (2012). 22744651 10.1002/mds.25058

[28] Selvadurai LP, Harding IH, Corben LA Cerebral abnormalities in Friedreich ataxia: a review. Neurosci. Biobehav. Rev. 84, 394–406 (2018). 28823857 10.1016/j.neubiorev.2017.08.006

[29] Khalil M, Teunissen CE, Otto M Neurofilaments as biomarkers in neurological disorders. Nat. Rev. Neurol. 14(10), 577–589 (2018). 30171200 10.1038/s41582-018-0058-z

[30] Zeitlberger AM, Thomas-Black G, Garcia-Moreno H Plasma markers of neurodegeneration are raised in Friedreich’s ataxia. Front. Cell Neurosci. 12, 366 (2018). 30425621 10.3389/fncel.2018.00366 PMC6218876

[31] Koeppen AH Friedreich’s ataxia: pathology, pathogenesis, and molecular genetics. J. Neurol. Sci. 303(1–2), 1–12 (2011). 21315377 10.1016/j.jns.2011.01.010 PMC3062632

[32] Koeppen AH, Morral JA, Davis AN The dorsal root ganglion in Friedreich’s ataxia. Acta Neuropathol. 118(6), 763–776 (2009). 19727777 10.1007/s00401-009-0589-x

[33] Morral JA, Davis AN, Qian J Pathology and pathogenesis of sensory neuropathy in Friedreich’s ataxia. Acta Neuropathol. 120(1), 97–108 (2010). 20339857 10.1007/s00401-010-0675-0

[34] Creigh PD, McDermott MP, Sowden JE *In-vivo* reflectance confocal microscopy of Meissner’s corpuscles in diabetic distal symmetric polyneuropathy . J. Neurol. Sci. 378, 213–219 (2017). 28566167 10.1016/j.jns.2017.05.025 PMC5791532

[35] Pagovich OE, Vo ML, Zhao ZZ Corneal confocal microscopy: neurologic disease biomarker in Friedreich ataxia. Annal. Neurol. 84(6), 893–904 (2018). 30294800 10.1002/ana.25355

[36] Smitt MS, Bird HA Measuring and enhancing proprioception in musicians and dancers. Clin. Rheumatol. 32(4), 469–473 23397145 10.1007/s10067-013-2193-7

[37] Proske U, Gandevia SC The proprioceptive senses: their roles in signaling body shape, body position and movement, and muscle force. Physiol. Rev. 92(4), 1651–1697 (2012). 23073629 10.1152/physrev.00048.2011

[38] Tsou AY, Paulsen EK, Lagedrost SJ Mortality in Friedreich ataxia. J. Neurol. Sci. 307(1–2), 46–49 (2011). 21652007 10.1016/j.jns.2011.05.023

[39] Hewer RL Study of fatal cases of Friedreich’s ataxia. Br. Med. J. 3(5619), 649–652 (1968). 5673214 10.1136/bmj.3.5619.649 PMC1986520

[40] Luijnenburg SE, Robbers-Visser D, Moelker A, Vliegen HW, Mulder BJ, Helbing WA Intra-observer and interobserver variability of biventricular function, volumes and mass in patients with congenital heart disease measured by CMR imaging. Int. J. Cardiovas. 26(1), 57–64 (2010). 10.1007/s10554-009-9501-y PMC2795153 19757150

[41] Kuruvilla S, Adenaw N, Katwal AB, Lipinski MJ, Kramer CM, Salerno M Late gadolinium enhancement on cardiac magnetic resonance predicts adverse cardiovascular outcomes in nonischemic cardiomyopathy: a systematic review and meta-analysis. Circ. Cardiovasc. Imaging. 7(2), 250–258 (2014). 24363358 10.1161/CIRCIMAGING.113.001144 PMC4007583

[42] Weidemann F, Liu D, Hu K The cardiomyopathy in Friedreich’s ataxia – new biomarker for staging cardiac involvement. Int. J. Cardiol. 194, 50–57 (2015). 26005806 10.1016/j.ijcard.2015.05.074

[43] Almutairi HM, Boubertakh R, Miquel ME, Petersen SE Myocardial deformation assessment using cardiovascular magnetic resonance-feature tracking technique. Br. J. Radiol. 90(1080), 20170072 (2017). 28830199 10.1259/bjr.20170072 PMC6047663

[44] Raman SV, Phatak K, Hoyle JC Impaired myocardial perfusion reserve and fibrosis in Friedreich ataxia: a mitochondrial cardiomyopathy with metabolic syndrome. Eur. Heart J. 32(5), 561–567 (2011). 21156720 10.1093/eurheartj/ehq443 PMC3106287

[45] Agarwal M, Shufelt C, Mehta PK Cardiac risk factors and myocardial perfusion reserve in women with microvascular coronary dysfunction. Cardiovasc. Diagn. Ther. 3(3), 146–152 (2013). 24282763 10.3978/j.issn.2223-3652.2013.08.01 PMC3839214

[46] Lodi R, Rajagopalan B, Blamire AM Cardiac energetics are abnormal in Friedreich ataxia patients in the absence of cardiac dysfunction and hypertrophy: an *in vivo* 31P magnetic resonance spectroscopy study. Cardiovasc. Res. 52(1), 111–119 (2001). 11557239 10.1016/s0008-6363(01)00357-1

[47] Bottomley PA, Panjrath GS, Lai S Metabolic rates of ATP transfer through creatine kinase (CK Flux) predict clinical heart failure events and death. Sci. Transl. Med. 5(215), 215re3 (2013). 10.1126/scitranslmed.3007328 PMC4440545 24337482

[48] Coelho-Filho OR, Shah RV, Mitchell R Quantification of cardiomyocyte hypertrophy by cardiac magnetic resonance: implications for early cardiac remodeling. Circulation 128(11), 1125–1133 (2013). 10.1161/CIRCULATIONAHA.112.000438 PMC5308548 23912910

[49] Vogel AP, Wardrop MI, Folker JE Voice in Friedreich ataxia. J. Voice. 31(2), 243.e9–243.e1 (2017). 10.1016/j.jvoice.2016.04.015 27501923

[50] Rosen KM, Folker JE, Vogel AP Longitudinal change in dysarthria associated with Friedreich ataxia: a potential clinical endpoint. J. Neurol. 259(11), 2471–2477 (2012). 22669353 10.1007/s00415-012-6547-x

